# Modeling the 2014 Ebola Virus Epidemic – Agent-Based Simulations, Temporal Analysis and Future Predictions for Liberia and Sierra Leone

**DOI:** 10.1371/currents.outbreaks.8d5984114855fc425e699e1a18cdc6c9

**Published:** 2015-03-09

**Authors:** Constantinos Siettos, Cleo Anastassopoulou, Lucia Russo, Christos Grigoras, Eleftherios Mylonakis

**Affiliations:** Applied Mathematics and Physical Sciences, National Technical University of Athens, Athens, Greece; Division of Genetics, Cell and Developmental Biology, University of Patras, Patras, Greece; Consiglio Nazionale di Ricerca, Napoli, Italy; Division of Infectious Diseases, Rhode Island Hospital, Warren Alpert Medical School of Brown University, Providence, Rhode Island, USA; Division of Infectious Diseases, Rhode Island Hospital, Warren Alpert Medical School of Brown University, Providence, Rhode Island, USA

**Keywords:** Agent-Based Simulations, Contact Network, ebola, EBOV, Effective reproductive number (Re), Equation-free approach, Forecasting

## Abstract

We developed an agent-based model to investigate the epidemic dynamics of Ebola virus disease (EVD) in Liberia and Sierra Leone from May 27 to December 21, 2014. The dynamics of the agent-based simulator evolve on small-world transmission networks of sizes equal to the population of each country, with adjustable densities to account for the effects of public health intervention policies and individual behavioral responses to the evolving epidemic. Based on time series of the official case counts from the World Health Organization (WHO), we provide estimates for key epidemiological variables by employing the so-called Equation-Free approach. The underlying transmission networks were characterized by rather random structures in the two countries with densities decreasing by ~19% from the early (May 27-early August) to the last period (mid October-December 21). Our estimates for the values of key epidemiological variables, such as the mean time to death, recovery and the case fatality rate, are very close to the ones reported by the WHO Ebola response team during the early period of the epidemic (until September 14) that were calculated based on clinical data. Specifically, regarding the effective reproductive number Re, our analysis suggests that until mid October, Re was above 2.3 in both countries; from mid October to December 21, Re dropped well below unity in Liberia, indicating a saturation of the epidemic, while in Sierra Leone it was around 1.9, indicating an ongoing epidemic. Accordingly, a ten-week projection from December 21 estimated that the epidemic will fade out in Liberia in early March; in contrast, our results flashed a note of caution for Sierra Leone since the cumulative number of cases could reach as high as 18,000, and the number of deaths might exceed 5,000, by early March 2015. However, by processing the reported data of the very last period (December 21, 2014-January 18, 2015), we obtained more optimistic estimates indicative of a remission of the epidemic in Sierra Leone, as reflected by the derived Re (~0.82, 95% CI: 0.81-0.83).

## INTRODUCTION

An outbreak of a communicable disease associated with a high fatality rate in the rural forest communities of Guinea in December 2013 has spiraled into an epidemic that is ravaging West Africa and evoking fear around the globe.^1^ Ebola virus (EBOV, formerly Zaire ebolavirus), one of the five species of the genus*Ebolavirus*, has been identified as the causative agent of this unprecedented epidemic, in terms of initial geographic occurrence, magnitude, complexity and persistence.^1^^,^ 2 EBOV was also involved in previous outbreaks in remote regions of Central Africa, with the largest, in the Democratic Republic of Congo (DR Congo, formerly Zaire) in 1976, accounting for merely 318 cases, including 280 deaths.^3^ The current Ebola Virus Disease (EVD) epidemic has plagued major urban centers in West Africa, in some of the most impoverished and logistically challenged countries of the world. According to the World Health Organization (WHO), as of January 12, 2015, 8,362 and 10,150 cases, including 3,556 and 3,067 deaths, respectively, have been officially reported in Liberia and Sierra Leone, the countries mostly afflicted along with Guinea, while the concern of further international spread has not ceased.^4^

Mathematical models are instrumental in providing guidance as to the future projections of such important ongoing public health crises, and in assessing the potential impact interventions might have towards transmission control.^5^ Models can be distinguished to four different categories associated with increasing levels of epidemiological realism and, consequently, with increasing difficulty in the systematic analysis:^5^^,^ 6 (i) Deterministic models in the form of differential or (integro)-partial differential equations are “continuum models” describing the coarse-grained dynamics of epidemics at the population level; (ii) relaxing the hypothesis of continuum models about infinite population, stochastic models incorporate stochastic parameters and variables; (iii) individual-based models with “memory” import the uniqueness of the individual behavior in a general population while relaxing the hypothesis of stochastic ones about structural uniformity in their interactions; (iv) the most complex, dynamic “social” network-based or agent-based models emphasize the heterogeneous nature and potential for delayed effects of the interactions between individuals.

Both deterministic and stochastic SEIR (susceptible-exposed-infectious-recovered) dynamic models have been used to study the 1995 and 2000 Ebola outbreaks in DR Congo and Uganda, which were caused by the Zaire and Sudan virus strains, correspondingly.^7^^,^ 8 Legrand *et al*.^9^, in particular, analyzed data from these two epidemics with a sixth-order stochastic compartmental model that incorporated explicitly the settings of transmission in the community, in the hospital and during traditional burial ceremonies. To explore the impact of control interventions, they simulated various epidemic scenarios and found that the rapid institution of control measures was a key parameter, whilst increasing hospitalization rate reduced the predicted epidemic size for both epidemic profiles.

For the current Ebola outbreak in West Africa, Rivers *et al.* 10 utilized the model proposed by Legrand^9^ to approximate and forecast the evolution of the spread in Liberia and Sierra Leone. Their model forecasted a continuously increasing epidemic until December 31, 2014, with medians of 117,877 and 30,611 cases for Liberia and Sierra Leone, respectively. Kiskowski^11^ combined a stochastic SEIR model with a three-scale community network model representing contacts between households and local communities, to demonstrate that the different regional trends of the early growth dynamics of the 2014 EBOV epidemic in Guinea, Sierra Leone and Liberia might be explained by disparate local community mixing rates. A compartmental stochastic, individual-based model employed by Gomes* et al.* 12 to approximate the dynamics of the Ebola outbreak worldwide at an early period of the outbreak, estimated a rapid increase of the cases in African countries, and a potential international threat on a longer time-scale.

Althaus^13^ used a deterministic SEIR dynamic model to estimate two vital epidemiological parameters for any infection, describing the spread of EBOV in West African countries, in this case: the basic and the effective reproduction numbers, *R_0_* and *R_e_*, correspondingly, i.e., the number of secondary cases generated by an infected index case in the absence and presence of control measures. This model indicated no decline in *R_e _* in Liberia until the end of August 2014, and a drop in *R_e _* in Sierra Leone to around unity, which would signal the termination of the epidemic, by the end of July 2014. Based on viral genetic sequencing data collected in an early period of the outbreak in Sierra Leone (until 18 June 2014), Stadler *et al.* 14 developed probabilistic SEIR models extending a birth-death model, as well as a deterministic coalescent model, and obtained median estimates of *R_0_* in the range of 1.65-2.18.

Herein, we investigate the epidemic dynamics of EVD in Liberia and Sierra Leone from May 27 to December 21, 2014, using an agent-based model whose dynamics evolve on small-world networks of sizes equal to the population of each country. The model takes into account the main epidemiological factors, including the effect of burial practices, to virus transmission. The most recent data (see Methods) are fitted to the model, employing the so-called Equation-Free approach^15^ , to obtain estimations of the expected structure and density of the underlying transmission network, the mean time from the onset of symptoms to death or to recovery, the case fatality rate and the per-contact transmission probability. Using the agent-based simulator, we also estimate the expected effective reproductive number *R_e_*. By monitoring the evolution of the epidemic parameters and network structure, we projected the evolution of the epidemic until early March 2015 (10 weeks after December 21). We show that the epidemic is at the stage of saturation in Liberia and, based on our analysis of publicly available data, we expect it to fade out by early March. On the contrary, in Sierra Leone the epidemic seems to be ongoing. However, an up-to-date analysis of the epidemic dynamics for Sierra Leone, revealed a significant change towards slower transmission rates in the very last period (i.e. between December 21, 2014-January 18, 2015) as reflected by the mean *R_e_* of around 0.82.

## METHODS

**Development of an agent-based model**

We have developed an agent-based model with \begin{equation*}\small{N}\end{equation*} individuals that interact through a small-world network constructed using the Watts & Strogatz (W&S) algorithm^16^ with a variable edge density. The edge density is defined as the number of links divided by the total possible links. In our model configuration, the density of connections can be adjusted at will. The construction of the network is based on random “rewiring” of links: starting with a ring network with \begin{equation*}k\end{equation*} neighbors per node ( \begin{equation*}\small{N\g\g k \g\g\ln{N} }\end{equation*} ) with probability \begin{equation*}\small{p_{rw}}\end{equation*} , an existent edge between a node and its first nearest neighbor (in a clock- or counter clock-wise sense) is cut and rewired with a randomly selected node. Self-connection or duplicate connections are not allowed. This process is repeated for each node and its first nearest neighbors. For \begin{equation*}\small{{p_{rw}=0}}\end{equation*} the initial ring is invariant, while for \begin{equation*}\small{p_{rw}=1}\end{equation*} the network is completely random. For intermediate values, \begin{equation*}\small{0 < p_{rw}<1}\end{equation*} , the network fluctuates between a regular and a random network, exhibiting the “small-world” property characterized by relatively small path lengths and high clustering coefficients. The network density denoted by “*α*” and defined as the ratio of the number of connections to the number of possible connections, is adjusted by randomly adding or subtracting the required number of links.

Agents can be in one of the following five discrete states:Susceptible \begin{equation*}\text{$(S)$}\end{equation*} , Exposed \begin{equation*}\text{$(E)$}\end{equation*} , Infected \begin{equation*}\text{$(I)$}\end{equation*} , Dead of the disease but not yet buried \begin{equation*}\text{$(D_{I})$}\end{equation*} , and Dead of the disease and safely buried \begin{equation*}\text{$(D_{b})$}\end{equation*} . The \begin{equation*}D_{I}\end{equation*} infectious state includes agents who die but whose burial entails risk for onward virus transmission. The contact network is denoted by \begin{equation*}\text{G(\bf{V}, \bf{E})}\end{equation*} , where \begin{equation*}{\bf{V}}=\left\{ v_{i} \right\}\text{ , i=1, 2, ..., N }\end{equation*} is the set of vertices corresponding to the \begin{equation*}N\end{equation*} agents, and \begin{equation*}\bf E\end{equation*} is the set of edges, i.e. the links between agents. An edge \begin{equation*}e_{v_{i}v_{j}}\end{equation*} is defined by \begin{equation*}\left{\bf{v}_{k}, \bf{v}_{l}}\end{equation*} , where \begin{equation*}\bf{v}_{k}, \bf{v}_{l}\in \bf{V}\end{equation*} are the agents associated with it; \begin{equation*}e_{v_{k}v_{l}} =1\end{equation*} if \begin{equation*}\left{\bf{v}_{k}, \bf{v}_{l}}\end{equation*} are connected, and \begin{equation*}e_{v_{k}v_{l}} =0\end{equation*} otherwise. Here, all links are bidirectional and self-contacts are not allowed, i.e. \begin{equation*}e_{v_{i}v_{i}} =0\end{equation*} . The neighborhood of agent \begin{equation*}v_k\end{equation*} is symbolized \begin{equation*}R_{v_{k}}\end{equation*} . The system’s state over the set of the nodes (edges) is represented by \begin{equation*}\text{Y(\bf{V})}\end{equation*} , where \begin{equation*}Y(v_k) \eq Y_{v_{k}}=\left{\text{S, E, I, D}_{\text{b}}, \text{D}_{\text{I}}, \text{R}\right}\end{equation*} is the set of the states of agent \begin{equation*}v_k\end{equation*} .

**Schematic of the Ebola virus infection states according to our proposed model.**
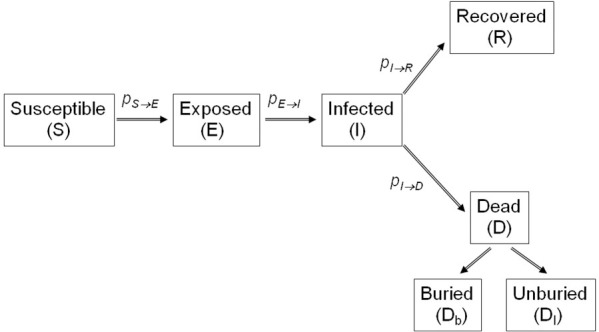
The shown probabilities reflect the rates of progression from one stage to the next. Agents dead of the disease that have not been buried yet ( \begin{equation*}D_I\end{equation*} ) remain potentially infectious, while virus transmission stops with dead agents that have been buried safely ( \begin{equation*}D_b\end{equation*} ). The inverse of the probability \begin{equation*}p_{E \rightarrow I}\end{equation*} that determines the rate by which an exposed agent becomes infectious, corresponds to the incubation period, i.e. the time from exposure to symptoms onset. This rate of incubation period is considered to be constant, set at \begin{equation*}p_{E \rightarrow I} = \frac{1}{9} \end{equation*} , as reported by the Who Ebola Response Team.^27^ The inverse of the probability \begin{equation*}p_{I \rightarrow D}\end{equation*} that determines the rate by which an infected agent dies of the disease, represents the time from symptoms onset to death.

The transition between states (also see Fig.1) is modelled as a discrete-time, discrete state *non-Markov random process *(hence, the dynamics incorporate “memory” in the transition between states), involving their own states and the states of their links. The governing rules that advance the system dynamics from time to time , with the unit of time propagation set to 1 day, are as follows:

\begin{equation*}\mathrm{p(Y_{v_k}(t+1)=D_b|Y_{v_k}(t-1)=D_I)=1}\end{equation*}

This first rule simply sets the time period from death to burial to two days, during which family members and loved ones may be infected due to physical contact with the dead, still-contagious body.

\begin{equation*}\mathrm{p}\left(\mathrm{Y_{v_{k}}(t+1)=E|Y_{v_{l}}(t)=I, Y_{v_{l}}(t)=D_I} \right)\mathrm{=p}_{\mathrm{S} \rightarrow  \mathrm{E}} , \boldsymbol{v}_l \in \boldsymbol{R}_{\mathrm{v_k}}\end{equation*}

The second rule implies that a susceptible agent gets exposed to the disease with a rate determined by the probability \begin{equation*}p_{S \rightarrow E}\end{equation*} per infected contact (still alive or dead, but not yet buried). Our model cuts the long-range links of a dead, potentially infectious agent, reflecting the fact that only relatives and close community members can be infected during unsafe funeral practices and rites.

\begin{equation*}
\mathrm{p(Y_{v_k} (t+1)=I|Y_{v_k} (t)=E)=p_{E \rightarrow I}}
\end{equation*}

The third rule implies that an exposed agent becomes infectious with a rate determined by the probability \begin{equation*}p_{E \rightarrow I}\end{equation*} , whose inverse corresponds to the incubation period, i.e. the time from exposure to symptoms onset.

Finally, an agent dies of the disease with a rate determined by the probability \begin{equation*}p_{I \rightarrow D}\end{equation*} (whose inverse is the time from symptoms onset to death):

\begin{equation*}
\mathrm{p(Y_{v_k} (t+1)=D_I |Y_{v_k} (t)=I)=p_{I \rightarrow D}}
\end{equation*}

Alternatively, an agent could recover with a rate determined by the probability \begin{equation*}p_{I \rightarrow R}\end{equation*} :

\begin{equation*}
p(Y_{v_k} (t+1)=R |Y_{v_k} (t)=I)=p_{I \rightarrow R}
\end{equation*}

Rules (4) and (5) define the case fatality rate, say \begin{equation*}p_{D/I}\end{equation*} , which is the ratio of deaths to the infected population.The above framework bypasses the need of derivation of closures for the emergent population-level equations, thereby providing a systematic, computationally strict approach for macroscopic-level analysis.

**Coarse-graining the agent-based dynamics for optimization and projection in time: the Equation-Free approach**

Over the past years it has been shown that a new multi-scale computational framework, namely the Equation-Free approach, can be used to establish synergism between “conventional” numerical analysis, system identification and optimization techniques, on one hand, and microscopic complex systems modeling, on the other. This computational methodology allows the extraction of “large-scale, system-level” information from very large-scale microscopic/stochastic simulators more efficiently than with current methods. Briefly, the Equation-Free methodology consists of the following steps:^15^^,^ 17 ^,^ 18 ^,^ 19 ^,^ 20 ^,^ 21

**(a)** Choose the statistics of interest for describing the long-term behavior of the system and an appropriate representation for them. This could be, for example, the average numbers of susceptible, exposed, infected, and recovered individuals. This continuum description is called \begin{equation*}\bf u\end{equation*} . These choices determine a restriction operator from the individual-based description \begin{equation*}\bf U\end{equation*} to the continuum description \begin{equation*}\mu =M \bf U\end{equation*} .

**(b)** Choose an appropriate lifting operator μ from the continuum description to the individual-based description \begin{equation*}\bf U\end{equation*} . For example, μ could make random node assignments, consistent with the continuum statistics. Note that \begin{equation*}\mu M = I\end{equation*} , that is, lifting from the continuum to the individual-based description, then restricting down again has no effect, apart from rounding-off effects.

**(c)** Prescribe a continuum initial condition \begin{equation*}\bf{u}({t_0})\end{equation*} .

**(d)** Transform this initial condition through lifting to one (or more) consistent individual-based realizations \begin{equation*}\bf{U}(t_0)=\mu \bf{u}(t_0)\end{equation*} .

**(e) **Evolve this (these) realization(s) using the individual-based model for a desired time \begin{equation*}T\end{equation*} , generating \begin{equation*}\bf{U}(T)\end{equation*} .

**(f)** Obtain the restrictions \begin{equation*}\bf{u}(T)=M\bf{U}(T)\end{equation*} .

Steps (a) to (f), constitute the *coarse time-stepper*, or as otherwise called the *coarse time-T map *that, given an initial coarse-grained state of the system \begin{equation*}\bf{u}_{t_k}\end{equation*} , \begin{equation*}\bf p\end{equation*} at time \begin{equation*}t_k\end{equation*} will report the result of the integration of the individual-based rules after a given time-horizon T (at time \begin{equation*}t_{k+1}\end{equation*} ), \begin{equation*}\text \bf{u}_{t_{k+1}}=\bf{\Phi}_{T}({\bf{u}_{t_k}} , {\bf p})\end{equation*} , where \begin{equation*}\text \bf{\Phi}_T  :  R^n\times  R^m \rightarrow R^n\end{equation*} having \begin{equation*}\bf {u_k}\end{equation*} as initial condition; \begin{equation*}\bf p\end{equation*} denotes the vector of the system parameters.

The *coarse time-stepper* can be also used to perform coarse projective integration to forecast the coarse-grained dynamics after a certain time horizon. The basic idea is that the coarse time-stepper can be used to approximate the time derivatives of the corresponding continuum formulation, even if the continuum equations are not known in closed form. Specifically, the following steps are executed:^15^

**(f)** Repeat step **(d)** over several time steps, giving several \begin{equation*}\bf{U}{(t_i)}\end{equation*} as well as their restrictions \begin{equation*}\text\bf{u}(t_i)=M\bf{U}(t_i) ,  i=1, 2, ..., k+1\end{equation*} **.**

**(g)** Use the chord connecting these successive time-stepper output points to estimate the derivative of the continuum variables. Note that this step does *not *require knowledge of the explicit continuum equations.

**(h)** Use this derivative in an outer integrator (such as an autoregressive (*ARX) *model) to estimate the continuum state \begin{equation*}\bf{u}(t_{k+1+m})\end{equation*} *much later in time.*

**(i) **Go back to step **(b)**.

The above framework bypasses the need of derivation of closures for the emergent population-level equations, hence providing a systematic, computationally strict approach for macroscopic-level analysis. Through appropriate calls of the agent-based simulator, one can estimate the same information that a continuum closed model would allow to evaluate if this model was available as an explicit formula. Using this framework, steady state and stability computations as well as projective integration (forecasting) and optimization of the complex-emergent dynamics can be performed in a fully computational manner, bypassing the need of analytical derivation of closures for the macroscopic-level equations. Thus, the methodology provides a closure on demand for the unavailable macroscopic dynamics. The hypothesis that has to be fulfilled for the implementation of the methodology is that a coarse-grained model for the dynamics at the macroscopic/continuum level in principle exists and closes in terms of a few coarse-grained variables, which are usually the low-order moments of the microscopically evolving distributions and simultaneously the apparent observables of the evolving phenomenon.

In this work, we demonstrate how the Equation-Free framework can be used to effectively analyze certain aspects of the dynamics of the agent-based Ebola virus epidemic simulator. It is assumed that the emergent dynamics can be effectively described by the zero-order moments of the evolving distributions, i.e. the expected values of \begin{equation*}
\left\{\mathrm{ S, E, I, D_{b}, D_{I}, R} \right\} \equiv \boldsymbol{u}
\end{equation*} . We employ the framework (a) to optimize the model parameters to fit the reported outbreak data, and, (b) to forecast the dynamics of the epidemic in the future. More specifically, two network characteristics and four epidemic rates are the model parameters to be fitted. The network characteristics are the switching rewiring probability for constructing the small-world network, \begin{equation*}p_{rw}\end{equation*} , and the density of the network, say \begin{equation*}d_G \eq a  \cdot  d_{G_0}\end{equation*} , which is adjusted at will by adding or subtracting the required number of links at a portion determined by the gaining factor \begin{equation*}a\end{equation*} ; \begin{equation*}d_{G_0}\end{equation*} is the density of the network constructed with the W&S algorithm \begin{equation*}
\mathrm{\left(  d_{G_0} = kN \right)}
\end{equation*} . Hence, the model parameters that are fitted are: \begin{equation*}p_{rw}\end{equation*} , \begin{equation*}a\end{equation*} , \begin{equation*}p_{D/I}\end{equation*} , \begin{equation*}p_{S \rightarrow E}\end{equation*} , \begin{equation*}p_{I \rightarrow D}\end{equation*} , \begin{equation*}p_{I \rightarrow R}\end{equation*} . The rate of incubation period is taken to be constant set as as \begin{equation*}p_{E \rightarrow I} = {1 \over 9}\end{equation*} reported by the Who Ebola Response Team.^21^

The expected averaged values of the agents’ states \begin{equation*}
Y(v_k) \equiv Y_{v_k} = \mathrm{\left\{S, E, I, D_{b}, D_{inf}, R\right\}}
\end{equation*} are computed over \begin{equation*}N_r = 8\end{equation*} network realizations, and \begin{equation*}N_s = 100\end{equation*} simulations for each one of the network realizations. A coarse-grained observable/product of the above representation is also the coarse-grained effective reproductive ratio, \begin{equation*}R_e\end{equation*} , defined as the average number of secondary infections produced by a typical infective person.

**Population estimates, outbreak data and the simulation process**

Based on the demographics reported by the United Nations (UN), the population estimates of Liberia and Sierra Leone are taken to be 4.2 and 6 million, respectively.^22^ Time series of the official case counts from the World Health Organization were used for model fitting.^4^ Case data, which included cumulative incidence and cumulative deaths by date of report for Liberia and Sierra Leone retrieved on 5^th^ of January, were found on Wikipedia^23^ and compiled from WHO case reports. These data sets, which do not distinguish between suspect, probable and laboratory-confirmed case counts, are considered to represent the best available estimates of the current state of the epidemic in the two severely afflicted West African countries.

Simulations were performed using May 27, 2014 as an initial date and a time horizon of 70 days (10 weeks) with an equal sliding window time interval; the last date was December 21, 2014. Thus, fitted values of the network and model parameters as well as estimates of the effective reproductive ratio were computed in sequences of succeeded time intervals of 70 days. The trust-region-reflective approach for nonlinear minimization was implemented for parameter estimation.^24^ We used Matlab as our simulation environment.^25^

## RESULTS

Figs. 2 & 4 show the cumulative numbers of infected and dead predicted by the model compared to the reported cases in Liberia and Sierra Leone, respectively. As shown, the proposed approach succeeds in approximating the reported data for both afflicted West African countries. Panels (a) of Figs. 3 & 5 depict the evolution of the estimated network characteristics, \begin{equation*}p_{rw}\end{equation*} and \begin{equation*}a\end{equation*} , while panels (b-e) portray the model parameters \begin{equation*}p_{D/I}\end{equation*} , \begin{equation*}p_{I \rightarrow R}\end{equation*} , \begin{equation*}p_{I \rightarrow D}\end{equation*} , and \begin{equation*}p_{S \rightarrow E}\end{equation*} that fit best to the reported Ebola virus epidemic dynamics in the two countries. The evolution of the estimated effective reproductive number, in Liberia and Sierra Leone is shown in panels (f) of Figs. 3 & 5, correspondingly.

**Simulation Results for Liberia from May 27 to December 21, 2014.**
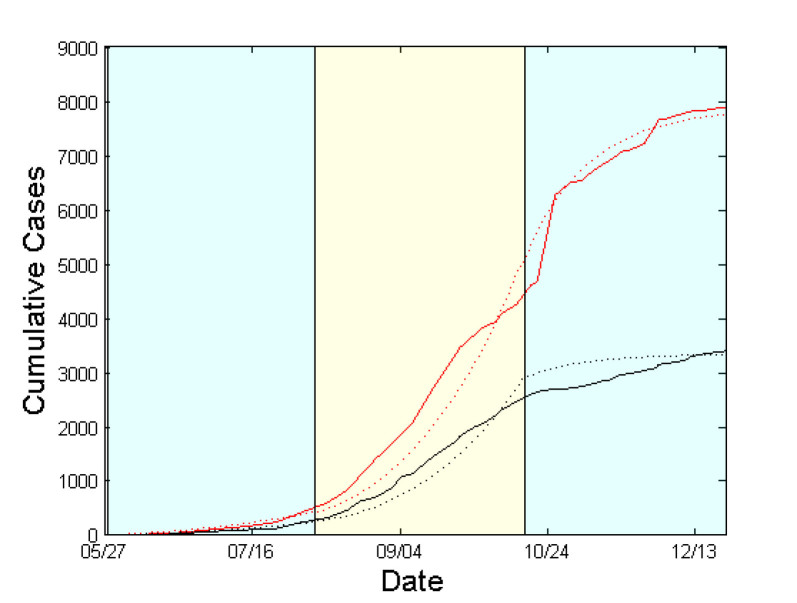
Expected cumulative cases of infected (dotted red) and dead (dotted black). WHO data are depicted by solid lines. The period under study has been tessellated into three windows with a length of 10 weeks each. For each window, the model parameters are estimated based on the data reported from WHO.

**The case of Liberia**

The contact network exhibits a rather random structure with a rewiring switching probability \begin{equation*}\left( p_{rw} \right) \end{equation*} of ~0.98 that remains constant during the whole period under study (Fig.3a). The density ratio of the network as represented by \begin{equation*}a\end{equation*} , on the other hand, appears to be constant, at ~0.70, during the first two study periods; then, in the period from mid October to December 21, it drops to ~0.55 (Fig.3a), reflecting a sparser network structure that can possibly be attributed to population isolation policies.

**Estimated model parameters for Liberia from May 27 to December 21, 2014.**
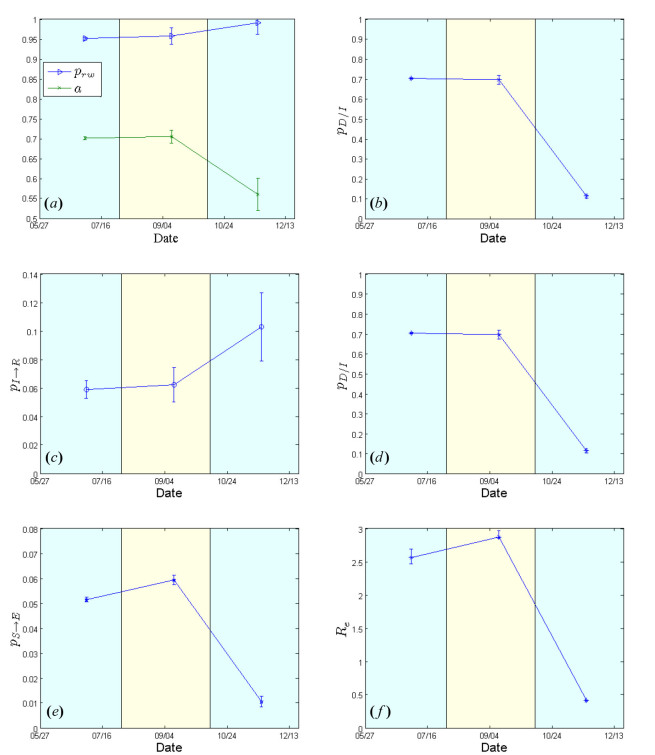
(a) Evolution of contact network characteristics, switching probability \begin{equation*}p_{rw}\end{equation*} , density ratio of the transmission network (a). (b) Case fatality rate \begin{equation*}p_{D/I}\end{equation*} . (c) 1/{recovery period} \begin{equation*}p_{I \rightarrow R}\end{equation*} . (d) 1/{period from inset of symptoms to death} \begin{equation*}p_{I \rightarrow D}\end{equation*} . (e) Per-contact transmission probability \begin{equation*}p_{S \rightarrow E}\end{equation*} . (f) Effective Reproductive number \begin{equation*}R_e\end{equation*} . 95% confidence intervals are also shown.

The case fatality rate \begin{equation*}\left( p_{D/I} \right) \end{equation*} that was estimated to be ~70% for the period extending from May 27 to mid October, dropped to ~10% subsequently (mid October-December 21) [Fig.3b]. The estimated recovery period (i.e. the inverse of \begin{equation*}p_{I \rightarrow R}\end{equation*} ) was ~17 days (until mid October) and ~10 days in the last study period (until December 21) [Fig.3c]. The corresponding values for the expected period from the onset of symptoms to death (i.e. the inverse of \begin{equation*}p_{I \rightarrow D}\end{equation*} ) were calculated to be ~8 days (May 27-mid October), and ~5 days in the last period (mid October-December 21) [Fig.3d]. The values of the per-contact transmission probability \begin{equation*}p_{S \rightarrow E}\end{equation*} , are shown in Fig.3e; they range from 0.05 (May 27-early August) to 0.01 most recently (mid October-December 21). The corresponding estimates of the effective reproductive number, \begin{equation*}R_e\end{equation*} , were ~2.5 (May 27-early August), rising to ~2.8 (early August-mid October), dropping sharply to ~0.42 in the last period (mid October-December 21) [Fig.3f], thus indicating a saturation of the epidemic.

In summary, using the expected values of model parameters, as computed in the last period, we estimate that if the reported data correspond to the real situation, the current epidemic should fade out in Liberia until early March 2015.

**Simulation Results for Sierra Leone from May 27 to December 21, 2014.**
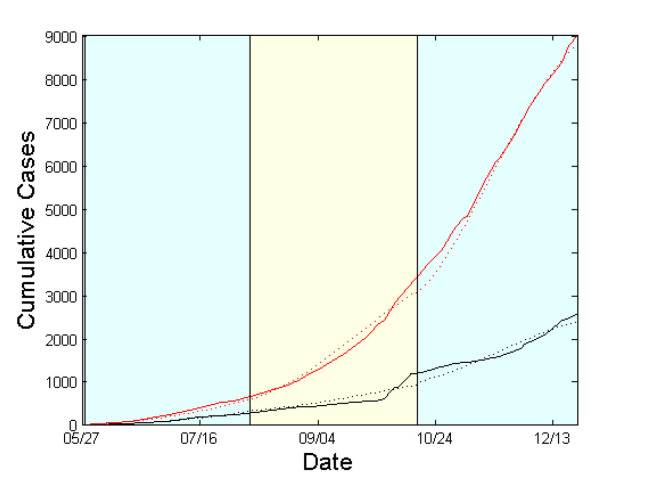
Expected cumulative cases of infected (dotted red) and dead (dotted black). WHO data are depicted by solid lines. The period under study has been tessellated into three windows with a length of 10 weeks each. For each window, the model parameters are estimated based on the data reported from WHO.

**The case of Sierra Leone**

Similarly to the case of Liberia, the contact network of Sierra Leone exhibits a rather random structure with a rewiring switching probability \begin{equation*}(p_{rw})\end{equation*} of ~0.98 that is kept constant during the study period (Fig.5a). The density ratio of the network as represented by \begin{equation*}a\end{equation*} appears to be ~0.70 during the first period (May 27-early August), dropping to ~0.58 in the last two periods (early August-December 21) [Fig.5a].

**Estimated model parameters for Sierra Leone from May 27 to December 21, 2014.**
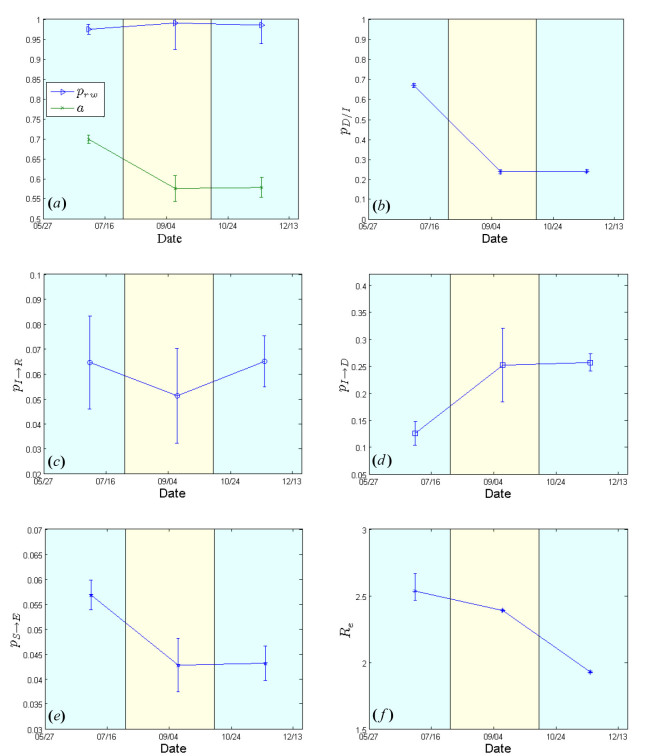
(a) Evolution of contact network characteristics, switching probability \begin{equation*}p_{rw}\end{equation*} , density ratio of the transmission network (a). (b) Case fatality rate \begin{equation*}p_{D/I}\end{equation*} . (c) 1/{recovery period} \begin{equation*}p_{I \rightarrow R}\end{equation*} . (d) 1/{period from inset of symptoms to death} \begin{equation*}p_{I \rightarrow D}\end{equation*} . (e) Per-contact transmission probability \begin{equation*}p_{S \rightarrow E}\end{equation*} . (f) Effective Reproductive number \begin{equation*}R_e\end{equation*} . 95% confidence intervals are also shown.

The case fatality rate \begin{equation*}(p_{D/I})\end{equation*} that was estimated to be ~67% for the period extending from May 27 to early August, dropped to ~24% from early September onwards (Fig.5b). The estimated recovery period (i.e. the inverse of \begin{equation*}p_{I \rightarrow R}\end{equation*} ) fluctuated slightly from ~15 days (May 27-early August) up to ~20 days (early August-mid October) and then returned to the originally estimated value of ~15 days in the last period (mid October-December 21) [Fig.5c]. The expected period from the onset of symptoms to death (i.e. the inverse of \begin{equation*}p_{I \rightarrow D}\end{equation*} ) was calculated to be ~8 days for the period between May 27 and early August, and ~4 days for the period between early August and December 21 (Fig.5d). Per-contact transmission probability values, \begin{equation*}p_{S \rightarrow E}\end{equation*} , dropped from 0.057 (May 27-early August) to ~0.043 from early August onwards (Fig.5e). The corresponding estimates of the effective reproductive rate, \begin{equation*}R_e\end{equation*} , were ~2.5 (May 27-early August), ~2.4 (early August-mid October) and ~1.9 in the last period (mid October-December 21) [Fig.5f], thereby indicating a still ongoing outbreak.

In order to forecast the evolution of the Ebola virus epidemic in Sierra Leone, we followed the coarse projection procedure described in the Methods. For that purpose, we extrapolated the values of the model’s parameters as estimated within the last two periods; the resulting parameter values were then fed to the simulator using as coarse initial conditions the values of \begin{equation*}
\mathrm{\left\{S, E, I, D_{b}, D_{I}, R\right\}}
\end{equation*} as computed on December 21. Based on the above procedure, we estimate that if the reported data correspond to the real situation, the expected cumulative number of infected cases may reach as high as 18,000 in early March, while the cumulative number of dead may reach as high as 5,000.

A summary of the estimated epidemiological parameters with their 95% confidence intervals for both countries is given in Table 1.

**Table 1. Key epidemiological features of the Ebola Virus Disease (EVD) epidemic in Liberia and Sierra Leone estimated by the model during the early, middle and last study periods (May 27-December 21, 2014).** p_rw_, Rewiring switching probability; CFR, Case fatality rate (p_D/I_); R_e_, Effective reproductive number

		Liberia		Sierra Leone	
Period	Variable	Mean	95% CI	Mean	95% CI
**Early**(May 27-August 4)	p_rw_	0.95	0.94-0.96	0.97	0.96-0.98
	Network density (α)	0.70	0.69-0.71	0.70	0.69-0.71
	Time to death (Days)	8.7	8.5-9.0	7.9	6.7-9.6
	Time to recovery (Days)	17	15.3-18.9	15.5	12.0-21.7
	CFR (%)	70.2	70.0-70.4	66.8	65.7-67.8
	R_e_	2.5	2.4-2.7	2.5	2.4-2.7
**Middle**(August 5-October 13)	p_rw_	0.96	0.93-0.98	0.99	0.92-1.0
	Network density (α)	0.70	0.69-0.72	0.57	0.54-0.61
	Time to death (Days)	8.0	7.0-9.3	4.0	3.1-5.4
	Time to recovery (Days)	16.0	13.5-19.8	19.6	14.2-30.9
	CFR (%)	69.5	67.2-71.8	24.0	22.9-25.0
	R_e_	2.9	2.8-3.0	2.4	2.3-2.5
**Last**(October 14- December 21)	p_rw_	0.99	0.96-1.0	0.98	0.94-1.0
	Network density (α)	0.56	0.52-0.60	0.58	0.55-0.60
	Time to death (Days)	5.0	4.3-6.0	3.9	3.7-4.1
	Time to recovery (Days)	9.7	7.9-12.7	15.4	13.3-18.2
	CFR (%)	11.5	10.5-12.5	24.1	23.5-24.8
	R_e_	0.42	0.41-0.43	1.93	1.92-1.94

***Update to the case of Sierra Leone***

Since the results we obtained by analyzing the reported data until December 21, 2014 showed that the epidemic was sustained in Sierra Leone, we decided to investigate further the current trends of the epidemic dynamics. Therefore, we expanded our analysis by taking into account the reported data for the country for the very last period (December 21, 2014-January 18, 2015). The results of this expanded analysis, which are indicative of a declining trend in the transmission potential of the virus, are shown in Table 2. Our analysis for this last period derived the following outcomes: rewiring switching probability \begin{equation*}(p_{rw}) \end{equation*} ~ 0.70 (95% CI: 0.61-0.78), density ratio of the network \begin{equation*}a\end{equation*} ~0.58 (95% CI:0.48-0.68), expected period from the onset of symptoms to death ~3.7 days (95% CI: 2.9-5.2 days), expected period from the onset of symptoms to recovery ~8.9 days (95% CI: 5.5-23 days), per-contact transmission probability ~0.03 (95% CI: 0.02-0.04), case fatality rate ~33% (95% CI: 30-35%), effective reproductive number, \begin{equation*}R_e\end{equation*} ~ 0.82 (95% CI: 0.81-0.83).

**Table 2. Updated key epidemiological features of the Ebola Virus Disease (EVD) epidemic in Sierra Leone estimated by the model by fitting the most recently available data (December 21, 2014-January 18, 2015).** p_rw_, Rewiring switching probability; \begin{equation*}\small p_{S \rightarrow E}\end{equation*} , Per contact transmission probability; CFR, Case fatality rate (p_D/I_); R_e_, Effective reproductive number

Period	Variable	Mean	95% CI
**Most recent** (December 21, 2014-January 18, 2015)	p_rw_	~0.70	0.61-0.78
	Network density (α)	~0.58	0.48-0.68
	Time to death (Days)	~3.7	2.9-5.2
	Time to recovery (Days)	~8.9	5.5-23
	p_S→E_	~0.03	0.02-0.04
	CFR (%)	~33	30-35
	R_e_	~ 0.82	0.81-0.83

## DISCUSSION

To approximate the dynamics of the current EVD epidemic in Liberia and Sierra Leone from an early date (May 27) to December 21, 2014, we propose the use of an agent-based model. The dynamics of the model evolve on a small-world network,^16^ the size of which matches the demographics of each country, while its density is adjustable to account for the impact of interventions. By exploiting the Equation-Free framework for multi-scale analysis,^15^ we estimated the evolution of the structure and the density of the EBOV transmission network that best fitted the data reported by WHO for the cumulative number of infected cases and deaths. Estimates for the major epidemiological parameters of the model were also derived using a sliding window of 10 weeks with an equal sliding step. Using this information, we also performed a projection in time and provide an estimate for the potential cumulative number of cases until early March 2015. The originality of our study stems from the fact that our model and analysis take into account the heterogeneity in the interactions of the agents within the network. This unique property is reflected on the plasticity of the resulting transmission network as well as on the variability of the network density, which changes over time in response to public health intervention policies and the behavioral responses of individuals as the epidemic evolves. The methodological approach succeeded in approximating the reported data of cumulative numbers of infected and dead in both afflicted West African countries.

For Liberia and Sierra Leone, our estimates of the effective reproductive number \begin{equation*}R_e\end{equation*} , which provides a measure of the potential of EVD transmission, as well as the estimates of the other key epidemiological variables, are quite close to the ones reported by the WHO Ebola Response Team and other groups. Transmissibility estimates from previous studies indicated an overall reproductive number \begin{equation*}R_o\end{equation*} ranging from 1.5 to 2.0 in West Africa in the early period of the epidemic.^12^^,^ 26 For Sierra Leone, in particular, the \begin{equation*}R_e\end{equation*} estimate of ~2.5 that we obtained in the early period of the epidemic (May 27-early August) agrees well with the estimated \begin{equation*}R_o\end{equation*} of ~2 to 2.5 given by the WHO Ebola Response Team and others.^13^^,^ 14 ^,^ 27 Rivers *et al.* 10 had calculated a lower \begin{equation*}R_o\end{equation*} of 1.78 for the same period in Sierra Leone. Our analysis revealed that \begin{equation*}R_e\end{equation*} remains well above 1 for the duration of the study period in Sierra Leone (~2.4 for the middle period from early August-mid October and ~1.9 for the last period extending from mid October to December 21). These values that are well above unity are indicative of an ongoing epidemic with a sustained transmission potential in Sierra Leone.

The situation appears to be different in the neighboring Liberia, where intervention policies seem to become effective after mid October, when a sharp decrease in new infections and deaths is observed. Accordingly, we estimated an of ~2.5 in the early period (May 27-early August), rising to ~2.9 in the middle period (early August-mid October) before dropping sharply to ~0.4 in the last study period (mid October-December 21). Chowell *et al.* 7 also reported a declining trend from 2.4 in early August, to 1.6 around September 6, to 1.3 by October 1 for the Liberian \begin{equation*}R_e\end{equation*} . Consistent with our early period estimate of ~2.5, \begin{equation*}R_e\end{equation*} values in the range of 2.2 to 2.5 were computed for that time interval by two other groups;^10^^,^ 28 others estimated \begin{equation*}R_e\end{equation*} to be lower, ranging from 1.6 to 1.8, during the early phase of the epidemic.^13^^,^ 27 For Liberia we estimate that the epidemic did not reach a saturation point before early October.

Additional notable differences in the estimated epidemiological profiles of the two countries from the early (May 27-early August) to the last study period (mid October- December 21) include: (i) nearly steady mean times to recovery from symptoms onset of ~15.4 days, with an intermediate increase to 19.6 days in the middle period (early August-mid October), in Sierra Leone* vs*. reduced from 17 to 9.7 days, with an intermediate value of 16 days, in Liberia; and (ii) lower reductions in the case fatality rate (from 66.8% to 24.1% in Sierra Leone* vs*. from 70.2% to 11.5% in Liberia) with dissimilar corresponding intermediate estimates of 24%*vs*. ~69.5%. Our estimates for the case fatality rate in the two countries are close to the numbers reported by the WHO Ebola response team for approximately the same period of reference (May 27-mid October); more specifically, we estimated the case fatality rate to be ~70.2% (95% CI, 70.0-70.4%) in Liberia and ~66.8% (95% CI, 65.7-67.8%) in Sierra Leone for this time period, while the WHO team had reported corresponding values of 72.3% (95% CI, 68.9-75.4) in Liberia and ~69.0% (95% CI, 64.5-73.1%) in Sierra Leone.^27^

Less pronounced differences between the two West African countries were found in the trends over time for the mean time from symptoms onset to death. In particular, for Liberia we estimated the mean time from symptoms onset to death to be about 9 days for the early period of May 27-mid October (the WHO team had reported a mean value of 7.9, yet with a large standard deviation), and 5 days for the last period from mid-October to December 21. For Sierra Leone, we estimated the mean time from symptom onset to death to be around 8 days for the early epidemic period from May 27 to early August (the WHO team had reported a mean value of 8.6, again with a large standard deviation), and around 4 days for the last period from mid-October to December 21.

At this point we should note that the findings of this work are subject to several limitations. The first and most important one is related to the quality and accuracy of the outbreak data that were fed to the proposed mathematical model compared to the real figures. Underreporting is certainly to be expected.^29^ Furthermore, as explained in the Methods, these data-sets do not distinguish between suspect, probable and laboratory-confirmed case counts. They are nonetheless considered to represent the best available estimates of the current state of the epidemic in the two severely afflicted West African countries.

We also performed a projection in time (10 weeks after December 21, 2014) of the EVD epidemic dynamics. Based on our approach, we estimate that the epidemic is already saturated in Liberia and it seems that it will be under control by March 2015. As reported recently,^30^ the movement and mixing of infected and uninfected patients in hospitals at the early stage of the outbreak was the driving force of the epidemic in Liberia.The subsequent decrease of incidence in the country is presumably attributable to the increasing availability of Ebola treatment units (which in turn contributed to drastically decreased hospital transmission), safe burials, and distribution of household protection kits.^30^ In contrast, the epidemic is sustained in Sierra Leone, with no evidence of slowing down at least until December 21, particularly as shown by the current effective reproductive number that remains well above unity. Based on the analysis before December 21, we predict that the cumulative number of cases in the country could reach as high as 18,000 and the cumulative number of deaths might exceed 5,000, by early March, if drastic control measures to curtail the epidemic are not taken. However, based on the analysis using the most recenlty reported data (December 21, 2014-January 18, 2015) our estimate for the effective reproductive number falls below unity (~0.82) indicating a different tendency compared to the previous period towards a decreased transmission potential in Sierra Leone. This decrease in \begin{equation*}R_e\end{equation*} is accompanied by a drop of an order of 28% in the switching rewiring probability of the transmission network rendering its structure more clustered, with less long-range connections between individuals.

The biological characteristics of the virus as well as the epidemiological features associated with its transmission are similar to those observed in previous outbreaks and, therefore, cannot account for the unprecedented scale of the current EVD epidemic in West Africa.^27^ The rapid institution of control measures (as detailed for example, in, Legrand *et al.* 9 and WHO,^27^)is key to halt the spread of such a deadly infection.
